# Alterations of Plasma Biochemical and Immunological Parameters and Spatiotemporal Expression of TLR2 and TLR9 in Gibel Carp (*Carassius auratus gibelio*) after CyHV-2 Infection

**DOI:** 10.3390/pathogens12111329

**Published:** 2023-11-08

**Authors:** Jinwei Gao, Yiwen Hu, Min Xie, Hao Wu, Jiayu Wu, Bingwen Xi, Rui Song, Dongsheng Ou

**Affiliations:** 1 Hunan Fisheries Science Institute, Changsha 410153, China; gaojinwei163@163.com (J.G.); xieminhaha@126.com (M.X.);; 2 Freshwater Fisheries Research Center of Chinese Academy of Fishery Sciences, Wuxi 214081, China; xibw@ffrc.cn; 3 Changsha Customs, Changsha 410000, China

**Keywords:** CyHV-2, gibel carp, biochemical indicators, TLR2, TLR9

## Abstract

Cyprinid herpesvirus II (CyHV-2), a highly contagious pathogen of gibel carp (*Carassius auratus gibelio*), causes herpesviral hematopoietic necrosis disease (HVHND) and enormous financial losses. However, there is limited information available regarding the changes in plasma biochemical and immunological parameters and the response characteristics of Toll-like receptor 2 (TLR2) and Toll-like receptor 9 (TLR9) in gibel carp after CyHV-2 infection. To address this knowledge gap, a sub-lethal CyHV-2 infection was conducted in gibel carp, and the sample was collected daily from 1 to 7 days post infection. The plasma biochemical analyses showed significant decreases in the content of glucose, total cholesterol (TCHO), and total protein (TP), along with marked increases in the level of uric acid, urea, creatinine (CREA), Complement 3 (C3), immunoglobulin D (IgD), and immunoglobulin M (IgM) as well as in the activity of alanine aminotransferase (ALT), aspartate transaminase (AST), alkaline phosphatase (ALP), and lactate dehydrogenase (LDH) in the infected group. Compared with the control group, the concentration of cortisol, triglyceride (TG), and Complement 4 (C4) had no noticeable alterations in the infected group. Real-time quantitative PCR analysis showed significant upregulation of TLR2 and TLR9 mRNA expression in the spleen, kidney, brain, liver, intestine, and gill post CyHV-2 infection. Interestingly, a time- and tissue-dependent expression profile has been comparatively observed for TLR2 and TLR9 in the above tissues of gibel carp after CyHV-2 infection, suggesting distinct roles between TLR2 and TLR9 in antiviral response to CyHV-2 infection. Overall, our results demonstrated that CyHV-2 infection led to the disruption of the physiological metabolic process and damage to the liver and kidney, and induced different spatiotemporal expression patterns of TLR2 and TLR9, ultimately stimulating antiviral response via innate and adaptive immune system. These findings may provide a deeper understanding of the host immunity response to CyHV-2 infection and offer novel perspectives for the prevention and treatment and therapeutic drug development against CyHV-2.

## 1. Introduction

Herpesviral hematopoietic necrosis disease (HVHND) has caused high mortality in gibel carp (*Carassius auratus gibelio*) and resulted in numerous economic losses in China since 2012 [1,2,3]. The causative agent, Cyprinid herpesvirus II (CyHV-2), is a linear double-stranded DNA (dsDNA) *Cyprinivirus* of the family Alloherpesviridae. CyHV-2, a pathogenic aquatic viral pathogen, causes high morbidity and mass mortality in all the different life stages of cyprinid species and the hybrids of goldfish and carps [4,5,6]. By far, HVHND cases have been reported throughout the world including Europe, North America, Oceania, and Asia [2]. Unfortunately, no effective vaccines and drugs are available for combating CyHV-2 infection to date; therefore, prevention of HVHND is essential to contend with this disease rather than treatment.

In fish, innate immunity is the first line of the immune defense system for pathogens’ invasion and clearance, and is the main way to protect themselves from harmful microbes [7,8]. The activation of the innate immune system is dependent on the recognition of pathogen molecules and subsequent activation of downstream signaling cascades [9]. Toll-like receptors (TLRs), a class of pathogen recognition receptors (PRRs), play the principal function in recognizing the pathogen-associated molecular patterns (PAMPs) present in diverse pathogens, including viruses, bacteria, fungus and their pathogenic constituents [10]. Unlike in mammals, where only 13 TLRs have been identified, at least 33 functional TLR types have been identified in teleost, which are grouped into 6 subfamilies [11]. TLRs have varying ligand specificity for different PAMPs, such as Toll-like receptor 2 (TLR2) and Toll-like receptor 9 (TLR9). Pioneer research on fish TLRs verified their analogous homology and functional characteristics with mammalian TLRs [12]. TLR2, a membrane-bound receptor located on the cell surface, can sense various microbial ligands, including lipoprotein, peptidoglycan, lipoteichoic acid of bacteria, envelope glycoproteins of viruses and glycosyl-phosphatidylinositol (GPI)-anchored proteins of parasites [13,14]. The broad spectrum of PAMPs recognized by TLR2 is owing to the formation of heterodimers with TLR1 subfamily members like TLR1 or TLR6, which expand its recognition ability and the range of ligands recognized [15]. On the other hand, TLR9, a receptor located in intracellular compartments, could detect unmethylated cytosine–phosphate–guanine DNA (CpG DNA) in fish and other aquatic vertebrates [16]. Previous studies have confirmed that TLR9 can recognize the unmethylated genomic DNA of pathogenic microbes, not the hypermethylated genomic DNA of aquatic life and mammals [10]. TLR2 and TLR9 have been identified in numerous fish species, such as *Carassius auratus* [17], *Siniperca chuatsi* [18], *Pelteobagrus fulvidraco* [19], and *Trachinotus ovatus* [20,21]. TLR2 and TLR9 in fish play crucial roles in the pathogen-induced immune response [9]. TLR2 and TLR9 response to CyHV-2 infection in gibel carp have been studied [1,22], but their time series and tissue-based expression during viral pathogenesis are unclear. Moreover, TLR localization is critical for ligand recognition and post-immune processes, whether these two receptors (TLR2 and TLR9) have differential functions in mediating the immunostimulatory activity of CyHV-2 infection in gibel carp has not been studied.

Hematological parameters are widely used for clinical diagnosis in vertebrates, and have a close relevance between the real-time health status of diseased vertebrates and causative microbes [23]. Blood parameters can accurately reflect the disease progress of fish during pathogen invasion. Previous studies have described the plasma indicators of moribund gibel carp naturally infected with CyHV-2 [1,24], but these studies were limited to certain time points and specific plasma indicators of viral infection. Moreover, little research has been carried out on metabolic enzymes, biochemical indicators, as well as innate and adaptive immune factors of gibel carp in plasma following experimental infection with CyHV-2. In this study, a sub-lethal concentration of CyHV-2 was injected into gibel carp to (1) evaluate biochemical and immunological parameters in plasma, (2) analyze gene expression of TLR2 and TLR9 gene in main tissues (brain, gill, liver, intestine, kidney, and spleen), and (3) comparatively investigate differential mRNA expression profiles of TLR2 and TLR9 gene in main tissues, aiming to provide novel insights into vaccine development, control, and prevention of HVHND.

## 2. Materials and Methods

### 2.1. Viral Infection and Sampling

A total of 120 healthy gibel carp (126.6 ± 23.2 g in weight and 18.9 ± 3.7 cm) were obtained from a concrete pond in the Changsha pilot research station of Hunan Fishery Sciences Institute, Changsha, China, and were free of the infection of parasites, bacteria, and viruses including CyHV-2 by our routine diagnostic procedures. Prior to the experiment, fish were acclimatized for 7 days in a 12.0 × 3.0 × 1.0 m^3^ aquarium at a water temperature of 25 ± 1 °C, pH 7.5, photoperiod of 12 h light/12 h dark, and dissolved oxygen concentration of 5.6 ± 1.0 mg/L in aerated freshwater. Fish were fed twice daily at 08:00 and 17:00 with the commercial diet at 3% body weight. Fish were randomly divided into the infected group and the control group (*n* = 60 per group), and equally loaded into 6 tanks (1.0 × 0.8 × 1.0 m^3^) to prevent crowding stress. This study is a continuation of our previous research work, and the details of virus isolation and viral infection were performed according to our previous study [25]. Briefly, the kidney and spleen were collected from naturally diseased gibel carp infected with CyHV-2. Tissues were homogenized with a mortar, pestled with sterile sand, and diluted 10-fold in M199 medium (containing 10% fetal bovine serum and bactericidal penicillin–streptomycin solution (10,000 U/mL)). Tissue homogenate was further disrupted by 3 cycles of freezing at −80 °C and thawing, and then centrifuged at 2057× *g* for 15 min at 4 °C. The resulting supernatant containing the CyHV-2 virus was filtered through a 0.22 μm membrane. To purify viruses and determine sublethal doses of CyHV-2, plaque formation and purification of CyHV-2 virus in cultured koi fin cells were carried out following the classical plaque forming unit (pfu) assay [26], and then well-isolated CyHV-2 virus was collected and verified by DNA analysis as described methods of Liang et al. [25]. As contamination prevention measures, sterile operation and disinfecting apparatus were applied in cell culture, the isolation and purification of CyHV-2, and the determination of sublethal doses of CyHV-2. Healthy fish in the infected group were injected intraperitoneally with 0.1 mL of CyHV-2 suspension. The virus used in the current study was derived from passage 3. Based on preliminary tests, 1 × 10^7^ pfu/mL of CyHV-2 suspension was employed to present the overall viral proliferation process and meet the demand for sampling amount throughout the experiment. A similar mock injection was given to the control group. During the viral infection experiment, fish of the infected group and control group were kept undisturbed in tanks with a water temperature of 25 ± 1 °C, dissolved oxygen concentration of 5.0 ± 1.0 mg/L, ammonia nitrogen concentration of 0.1 ± 0.05 mg/L, and photoperiod of 12 h light/12 h dark, and were fed to satiation with the commercial diet twice a day (8:00 and 17:00). To minimize stress on fish and ensure optimal welfare, fish of the infected group and control group (*n* = 6 per day; 42 total) were anesthetized firstly using MS-222 (50 mg/L) at 1, 2, 3, 4, 5, 6, and 7 days post infection, and blood samples were rapidly collected from the caudal vein following the described method [27]. Subsequently, brain, liver, kidney, spleen, intestine, and gill fragments of each group (*n* = 6) were dissected individually, frozen immediately in liquid nitrogen, and then kept at −80 °C for RNA extraction (see below Section 2.3). Plasma was separated by centrifugation (3000× *g*, 4 °C, 10 min), and then stored at −80 °C until further use. 

### 2.2. Measurements of Biochemical and Immunological Parameters in Plasma

Commercial detection kits of glucose (Catalogue Number (Cat. No.): 105-000495-00), alanine aminotransferase (ALT, Cat. No.: 105-000477-00), aspartate transaminase (AST, Cat. No.: 105-000478-00), alkaline phosphatase (ALP, Cat. No.: 105-000444-00), lactate dehydrogenase (LDH, Cat. No.: 105-000481-00), total cholesterol (TCHO, Cat. No.: 105-000477-00), total protein (TP, Cat. No.: 105-015579-00), uric acid (Cat. No.: 105-001423-00), triglyceride (TG, Cat. No.: 105-000484-00), creatinine (CREA, Cat. No.: 105-000457-00), urea (Cat. No.: 105-000487-00), immunoglobulin D (IgD, Cat. No.: 105-001432-00), and immunoglobulin M (IgM, Cat. No.: 105-001434-00) were purchased from Mindray Medical International Ltd. (Shenzhen, China) and assayed using Mindray BS-400 automatic biochemical analyzer (Mindray Medical International Ltd., Shenzhen, China). Commercially available kits of Complement 3 (C3, Cat. No.: E032-1-1), complement 4 (C4, Cat. No.: E032-1-1), and cortisol (Cat. No.: H094-1-1) were purchased from Nanjing Jiancheng Bioengineering Institute (Nanjing, China) and determined using a Multiskan GO spectrophotometer (Thermo Fisher Scientific, Waltham, MA, USA). All commercial kits were in use following the manufacturer’s protocols.

### 2.3. RNA Extraction, cDNA Synthesis, and Quantitative Real-Time PCR

Total RNA was extracted from the above-mentioned tissues using RNAiso Plus (Takara Biomedical Technology Co., Ltd., Beijing, China) conforming to classic protocols. First-strand cDNA was synthesized using a PrimeScript™ RT reagent Kit with gDNA Eraser (Takara Biomedical Technology Co., Ltd., Beijing, China) following the manufacturer’s protocol. Quantitative real-time PCR was performed on a LightCycler^®^ 96 system (F. Hoffmann-La Roche Ltd., Mannheim, Germany) with the TB Green dye-based detection method. The primers used in this study are listed in Table 1, and molecular characterization of TLR2 and TLR9 are summarized in Table 2 based on the sequence analysis methods of Sudhagar et al. [28]. Amplification reactions were performed with a volume of 25 μL containing 2 μL cDNA (ng/μL), 12.5 μL 2 × TB Green Premix Ex Taq (Takara Biomedical Technology Co., Ltd., Beijing, China), 0.5 μL forward primer (10 μM), 0.5 μL reverse primer (10 μM), and 9.5 μL RNase-free water. The reaction included an initial denaturation step of incubation for 30 s at 95 °C, followed by 40 cycles of denaturation at 95 °C for 5 s, annealing at 58 °C for 30 s, and extension at 72 °C for 30 s. Triplicates of each sample and endogenous control were amplified. The housekeeping gene 18S rRNA was utilized as an internal control for cDNA normalization. Relative quantification was calculated using the 2^−ΔΔCt^ method [1].

### 2.4. Statistical Analysis

Data are depicted as the mean ± standard deviation ($\bar{x}$ ± S.D). Significant differences between sample means of the infected group and control group were tested using one-way ANOVA via SPSS version 20.0 for Windows. Differences were measured and considered to be statistically significant at *p* < 0.05, but not significant at *p* > 0.05. All figures were graphed using the GraphPad Prism 8.3 software (https://www.graphpad.com/ (accessed on 15 October 2019), RRID: SCR_002798).

## 3. Results

### 3.1. Clinical Signs and Mortality of Gibel Carp after CyHV-2 Infection

**Figure 1:**
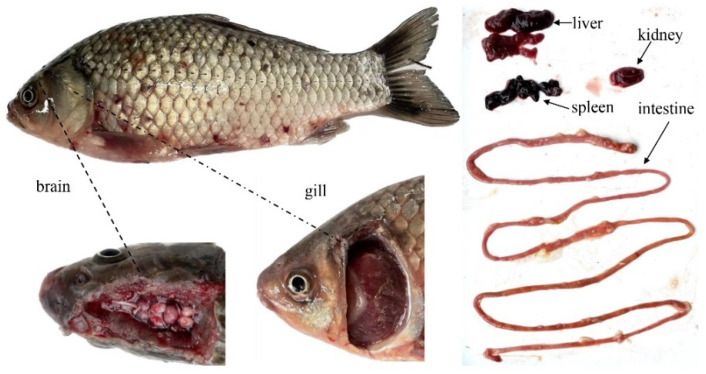
Clinical signs of main tissues of infected gibel carp. Gibel carp in infected group presented hyperemia in the regions of the submaxilla, abdomen, and liver, brain hyperemia, gill hemorrhages, splenomegaly, renal hypertrophy, and intestine swelling.

**Figure 2:**
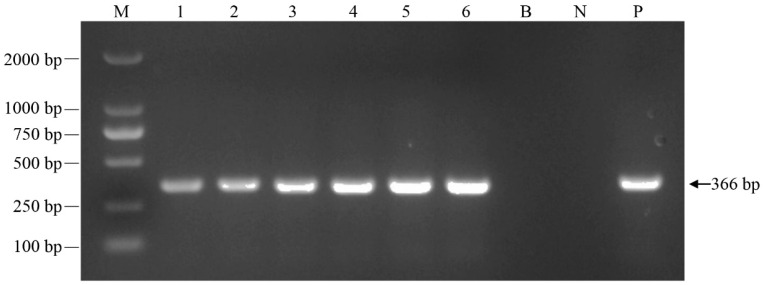
PCR products of helicase gene of CyHV-2 in six tissues of infected gibel carp. bp: base pair; lane M: DL 2000 DNA marker; lane B: no template as blank control; lane N: DNA template extracted from spleen of healthy gibel carp as negative control, lane P: DNA template extracted from CyHV-2 suspension as positive. Lane 1–6: DNA template extracted from brain, gill, liver, kidney, intestine, and spleen, respectively.

At 1 and 2 days post infection (dpi), no clinical symptoms of HVHND were observed in the infected group. At 3, 4, 5, 6, and 7 dpi, gibel carp showed typical clinical signs of HVHND including the hyperemia of submaxilla, abdomen, and liver, brain hyperemia, gill hemorrhages, splenomegaly, renal hypertrophy, and intestine swelling (Figure 1). Gibel carp began to die at 3 dpi and mortality continued until 7 dpi, causing a cumulative death rate of 25%. Among the control group, no fish showed any clinical signs of HVHND and mortality throughout the experimental period. Moreover, the CyHV-2 isolation and confirmation from infected gibel carp were conducted by referring to the previous method [29]. As shown in Figure 2, PCR examination of tissue samples (brain, gill, liver, kidney, spleen, and intestine) from infected gibel carp were found to be positive (366 bp of helicase gene), suggesting that CyHV-2 infection had a direct correlation with morbidity and mortality of gibel carp in the infected group.

### 3.2. Changes of Biochemical Parameters in Plasma of Gibel Carp

**Figure 3:**
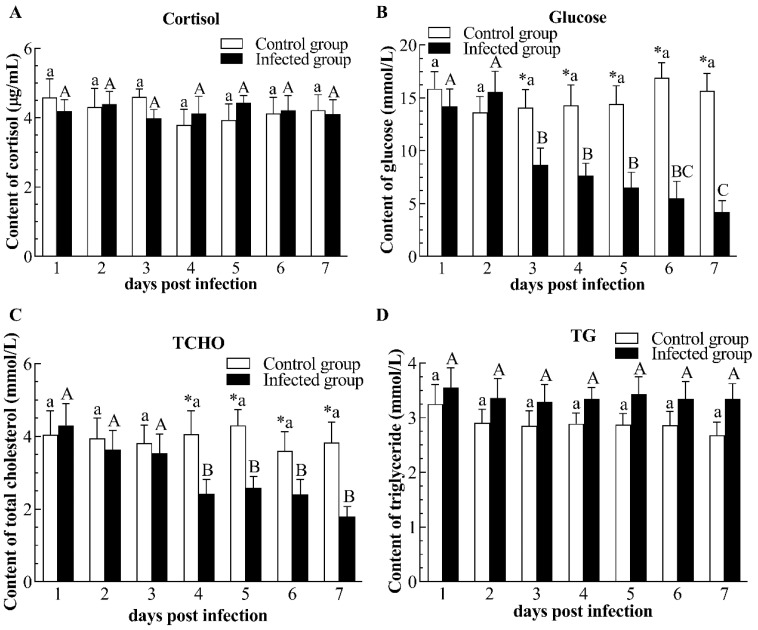
Content of cortisol (**A**), glucose (**B**), total cholesterol (**C**), and triglyceride (**D**) in plasma of the infected and healthy gibel carp. TCHO: total cholesterol; TG: triglyceride. The asterisk (*) indicates significant differences at the same point of time between the infected group and control group (*n* = 6, *p* < 0.05); bars with different upper and small capital letters at different points of time have significant differences in control group and infected group (one-way ANOVA, *p* < 0.05).

**Figure 4:**
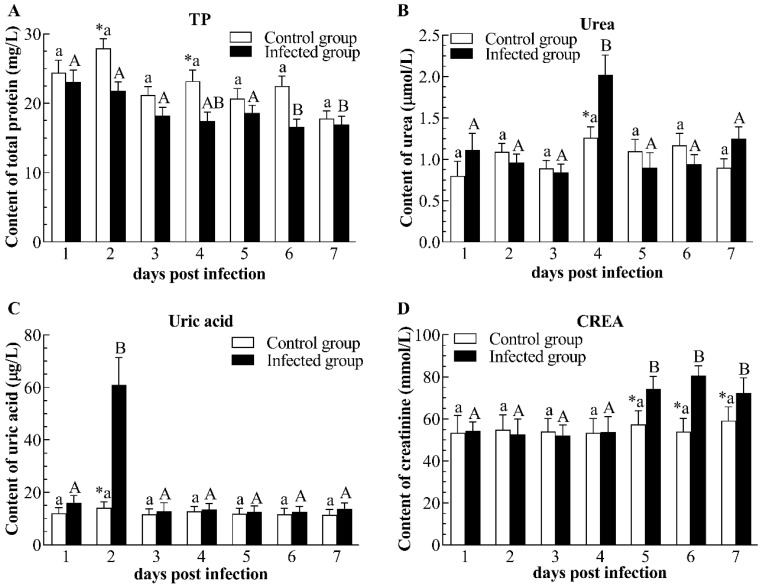
Content of total protein (**A**), urea (**B**), uric acid (**C**), and creatinine (**D**) in plasma of the infected and healthy gibel carp. TP: total protein; CREA: creatinine. The asterisk (*) indicates significant differences at the same point of time between the infected group and control group (*n* = 6, *p* < 0.05); bars with different upper and small capital letters at different points of time have significant differences in control group and infected group (one-way ANOVA, *p* < 0.05).

**Figure 5:**
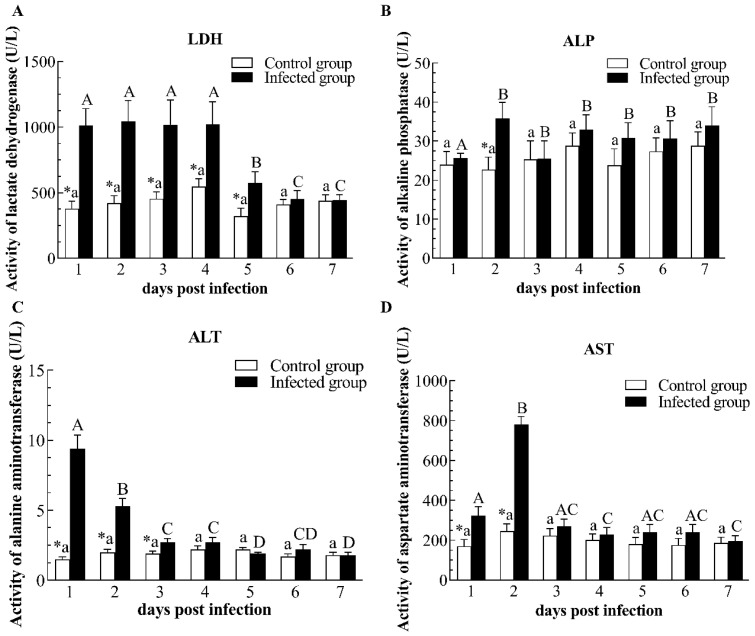
The enzyme activities of lactate dehydrogenase (**A**), alkaline phosphatase (**B**), alanine aminotransferase (**C**), and aspartate aminotransferase (**D**) in plasma of the infected and healthy gibel carp. LDH: lactate dehydrogenase; ALP: alkaline phosphatase; ALT: alanine aminotransferase; AST: aspartate transaminase. The asterisk (*) indicates significant differences at the same point of time between the infected group and control group (*n* = 6, *p* < 0.05); bars with different upper and small capital letters at different points of time have significant differences in control group and infected group (one-way ANOVA, *p* < 0.05).

CyHV-2 infection had no significant influence on the content of cortisol and TG when compared to the control group (Figure 3A,D). However, CyHV-2 infection led to a remarkable reduction in glucose concentration from 3 to 7 dpi, showing a trend of continuous decrease (Figure 3B). Similarly, the level of TCHO significantly decreased from 4 to 7 dpi in the infected group (Figure 3C). In terms of TP concentration, CyHV-2 infection caused a notable increase at 2 and 4 dpi (Figure 4A). Likewise, an increment of urea and uric acid content in infected fish was observed at 4 and 2 dpi, respectively (Figure 4B,C). Compared with the control group, CyHV-2 infection elevated CREA content at 5–7 dpi (Figure 4D). In addition, plasma LDH activity in infected gibel carp significantly rose at 1–5 dpi (Figure 5A). Compared with non-infected fishes, the plasma ALP activity of infected fishes obviously increased at 2 dpi (Figure 5B). Moreover, plasma ALT activity of infected fishes sharply increased at 1–3 dpi when compared to non-infected fishes, then decreased to the control group’s level (Figure 5C). Plasma AST activity of the infected group was significantly elevated at 1 and 2 dpi as compared with controls, but decreased to control levels at 3 dpi (Figure 5D).

### 3.3. Changes in Immunological Parameters in Plasma of Gibel Carp

**Figure 6:**
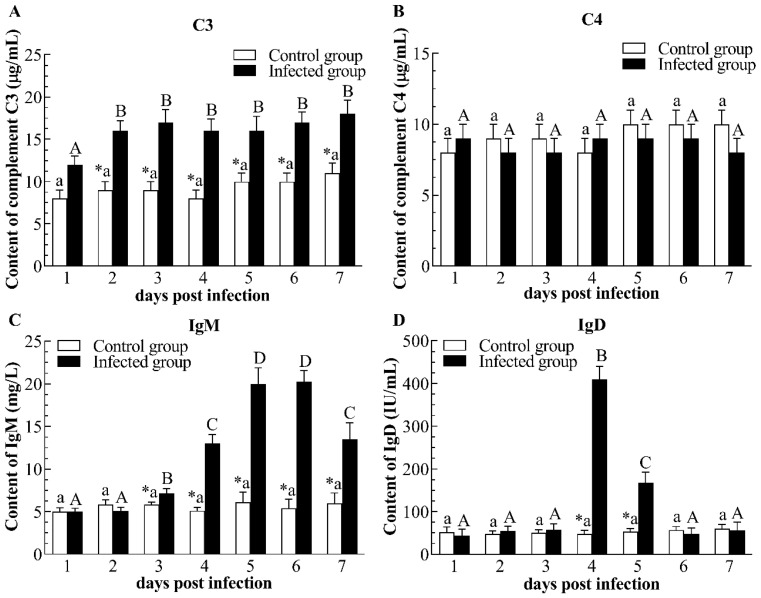
Content of complement 3 (**A**), complement 4 (**B**), IgM (**C**), and IgD (**D**) in plasma of the infected and healthy gibel carp. C3: Complement 3; C4: Complement 4; IgD: immunoglobulin D; IgM: immunoglobulin M. The asterisk (*) indicates significant differences at the same point of time between the infected group and control group (*n* = 6, *p* < 0.05); bars with different upper and small capital letters at different points of time have significant differences in control group and infected group (one-way ANOVA, *p* < 0.05).

Compared with the control group, the augment of plasma C3 concentration was induced at 2–7 dpi following CyHV-2 infection (Figure 6A). By contrast, the plasma C4 concentration of the infected group was not statistically significant throughout the trial when compared to the control group (Figure 6B). In comparison to the control group, the level of IgM in the infected group increased firstly at 3 dpi, and showed incremental augment at 5 and 6 dpi, then decreased slightly at 7 dpi, but its level was higher than that of controls (Figure 6C). The level of IgD in the infected group had marked enhancement at 4 and 5 dpi when compared to the control group (Figure 6D).

### 3.4. Gene Expression of TLR2 in Gibel Carp after CyHV-2 Infection

**Figure 7:**
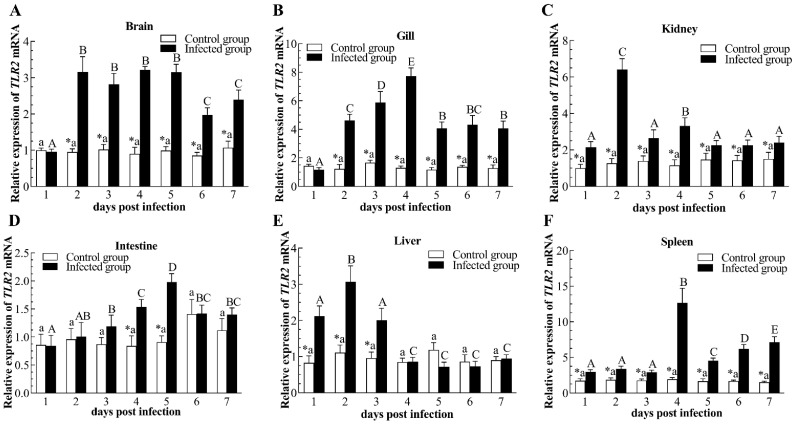
Relative expression of Toll-like receptor 2 (TLR2) in brain (**A**), gill (**B**), kidney (**C**), intestine (**D**), liver (**E**), and spleen (**F**) of the infected and healthy gibel carp. The asterisk (*) indicates significant differences at the same point of time between the infected group and control group (*n* = 6, *p* < 0.05); bars with different upper and small capital letters at different points of time have significant differences in control group and infected group (one-way ANOVA, *p* < 0.05).

In comparison with the control group, remarkable up-regulation of TLR2 transcripts was observed at 2–7 dpi in the brain and gill after viral infection, reaching a peak value of the control group at 3 dpi (3.58-fold) and 4 dpi (5.95-fold), respectively (Figure 7A,B). TLR2 expression levels in the kidney and spleen increased notably throughout the CyHV-2 infection trial, with the highest expression at 2 dpi (5.05-fold) and 4 dpi (6.55-fold), respectively (Figure 7C,F). However, fish infected with CyHV-2 showed higher TLR2 transcripts in the intestine at 4–5 dpi with respect to the control group (*p* < 0.05), with the peak value reaching 2.18-fold at 5 dpi (Figure 7D). After CyHV-2 infection, TLR2 transcripts in the liver raised rapidly at 1 dpi, peaked at 2 dpi, and up-regulated noticeably at 3 dpi, then returned to the control level from 4 to 7 dpi (Figure 7E).

### 3.5. Gene Expression of TLR9 in Gibel Carp after CyHV-2 Infection

**Figure 8:**
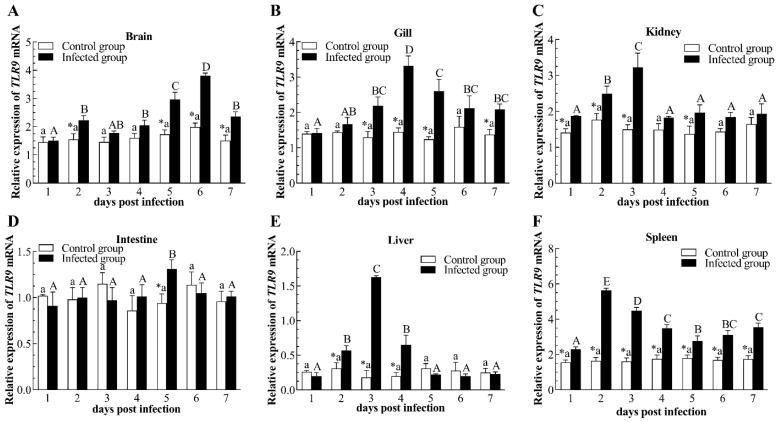
Relative expression of Toll-like receptor 9 (TLR9) in brain (**A**), gill (**B**), kidney (**C**), intestine (**D**), liver (**E**), and spleen (**F**) of the infected and healthy gibel carp. The asterisk (*) indicates significant differences at the same point of time between the infected group and control group (*n* = 6, *p* < 0.05); bars swith different upper and small capital letters at different points of time have significant differences in control group and infected group (one-way ANOVA, *p* < 0.05).

TLR9 expression levels in different tissues are shown in Figure 8. In the brain, expression levels of TLR9 markedly up-regulated at 2, 5, 6, and 7 dpi, reaching a peak value 1.91-fold that of the control group at 6 dpi (Figure 8A). Similar up-regulation of TLR9 transcripts was observed in the gill after infecting with CyHV-2 at 3, 4, 5, and 7 dpi, reaching a peak value 2.29-fold that of the control group at 4 dpi (Figure 8B). After CyHV-2 infection, TLR9 expression levels in the kidney showed a gradual augment, and had a maximum value at 3 dpi, then recovered to the control level at 4, 6, and 7 dpi (Figure 8C). However, TLR9 transcripts in the intestine did not show statistical change, except at 5 dpi following CyHV-2 infection (Figure 8D). In the liver, mRNA expression levels of TLR9 substantially increased from 2 dpi to 4 dpi and had a crest value at 3 dpi, then returned to the control level from 5 dpi to 7 dpi (Figure 8E). Intriguingly, TLR9 expression levels in the spleen notably raised throughout the CyHV-2 infection trial, with the highest expression reaching 3.44-fold that of the control group at 2 dpi (Figure 8F).

### 3.6. Differential mRNA Expression Profiles between TLR2 and TLR9 of Gibel Carp Post CyHV-2 Infection

**Figure 9:**
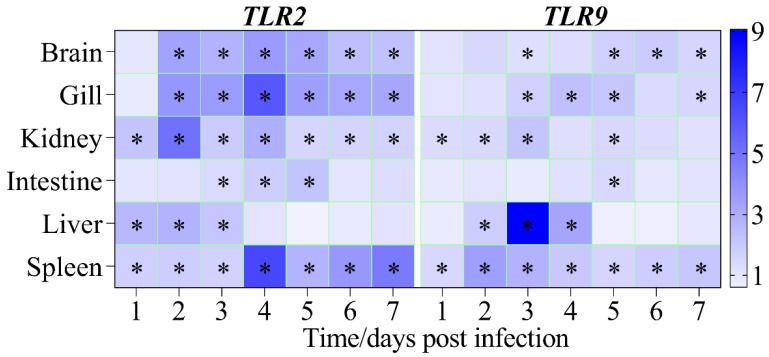
Heatmap showing fold induction of Toll-like receptor 2 (TLR2) and Toll-like receptor 9 (TLR9) gene expression between infected gibel carp and healthy gibel carp following normalization to 18S rRNA at the same time point. Significant differences in the relative fold change between the infected group and control group at each time points are indicated with an * (*p* <0.05).

As shown in Figure 9, TLR2 in the brain and gill displayed higher levels of fold induction from 2 dpi to 7 dpi than that of TLR9 in the brain and gill at the given time point. Throughout the experimental period, both TLR2 and TLR9 in the spleen continuously overexpressed at a higher level, being highest at 4 dpi and 2 dpi, respectively. In the kidney, TLR2 showed enhanced induction of gene expression levels throughout the experiment, whereas TLR9 showed significant induction of gene expression levels at 1, 2, 3, and 5 dpi. Likewise, TLR2 transcripts in the liver were significantly stimulated at 1–3 dpi, whereas TLR9 transcripts in the liver showed notable upregulation at 2–4 dpi. Moreover, TLR2 in the intestine showed a reinforced increment of gene expression level at 3–5 dpi; however, TLR9 in the intestine only showed increased fold induction at 5 dpi. Except in the liver, the maximum fold change of TLR2 is much higher than that of TLR9 in the brain (3.58-fold vs 1.91-fold), gill (5.95-fold vs 2.29), kidney (5.05-fold vs 2.15-fold), intestine (2.18-fold vs 1.39-fold), and spleen (6.55-fold vs 3.44-fold).

## 4. Discussion

Crucian farming is one of the most important sectors in aquaculture and of major importance to food security. CyHV-2 has been identified as the pathogeny of HVHND with high pathogenicity and mortality, threatening crucian aquaculture worldwide. During the progress of an infection, biochemical and immunological parameters of plasma are commonly used to monitor health status and physiological changes in fish [23]. Glucose is the main substrate of energy metabolism in plasma, and its content is in the dynamic balance in fish. In our study, the level of glucose in plasma descended continuously upon infection with CyHV-2. Similar lower glucose levels in plasma have been observed in various species of fish following pathogens’ infection [24,30]. Plasma TP plays crucial roles in osmotic pressure maintenance and metabolite transport, and its levels are a reflection of nutritional metabolism and non-specific immunological function [31]. In the current study, CyHV-2 infection led to descending TP content of plasma in gibel carp, which implies the deterioration in protein metabolism and non-special immunity. A similar downtrend of TCHO has also been observed in plasma during the course of CyHV-2 infection. TCHO is an important part of fish lipometabolism and is closely correlated with their nutritional condition. Therefore, our results suggest that CyHV-2 infection disrupted lipid metabolism, resulting in a disturbance of lipid homeostasis. Pioneer investigations have noted that the content of glucose, TP, and TCHO is positively related to the function of material energy metabolism and the ability to fight off pathogens’ infection [23]. Combined with the above results, the disruption of metabolic homeostasis of glucose, protein, and lipid caused by CyHV-2 infection reduces the ability to resist disease in gibel carp. Urea, uric acid, and CREA are the metabolites of lipid and protein catabolism, and can therefore be used as important physiological indicators of renal function [32]. Our results showed that the plasma content of urea, uric acid, and CREA significantly increased in gibel carp after CyHV-2 infection, indicating that the kidney of infected gibel carp has been substantially lesioned. Consulting the previous work, the reduction in plasma TP content also indicated that CyHV-2 infection damaged the kidneys of gibel carp [33]. As is known, the activity of AST and ALT in plasma is very low under normal circumstances, and therefore the increased activity of ALT and AST in plasma could be the most specific and widely used biomarkers of hepatic injury [34]. In this study, the activity of ALT and AST sharply elevated during the early stages of CyHV-2 infection (1–2 dpi and 1–3 dpi, respectively), which indicated CyHV-2 infection led to acute liver damage. LDH is an important enzyme family in anaerobic glycolysis and gluconeogenesis. ALP, a group of metabolic enzymes, regulates calcium and phosphorus metabolism and phosphate group transfer, playing a key role in the absorption and utilization of nutrients. Like AST and ALT, ALP and LDH are mainly present in hepatic cells; thus, their increased activity in plasma indicates liver injury. Here, gibel carp of the infected group showed higher enzyme activity of plasma ALP and LDH, indicating that CyHV-2 infection harmed the liver function and lesioned liver tissue of gibel carp. LDH, ALP, AST, and ALT in plasma can therefore be used as diagnostic biomarkers of acute liver damage in fish after CyHV-2 infection. Similarly, urea, uric acid, and CREA in plasma can be regarded as diagnostic biomarkers of kidney injury in fish after CyHV-2 infection. On the other hand, kidney and liver injury of gibel carp, inferred by plasma biochemical indicators (such as LDH, AST, ALT, CREA, and urea), matched the preliminary histopathological findings [3]. Consequently, plasma biochemical indicators of fish are the notable biomarkers for indicating pathological injury of tissue or organ and the progress of infectious diseases.

The immune system of teleost fishes, composed of innate and acquired immune systems, is the crucial protective barrier responsible for sensing and eliminating invading pathogens. C3, the highest content of complement components in plasma, performs vital roles in the activation of the innate immune system by promoting phagocytosis and neutralization of viruses [35]. Apart from its well-known functions in innate immunity, C3 also acts as a bridge to adaptive immunity, provoking a robust and rapid response against microorganisms [36]. In the present study, plasma C3 content significantly elevated from 2 dpi to 7 dpi after CyHV-2 infection, indicating that the non-specific immune system represented by C3 was activated by CyHV-2 and created the physiological condition for subsequent activation of the acquired immune system. IgM and IgD, as the key immune factors to assess the acquired immune response of fish, are involved in the pathological process of infectious diseases and play pivotal functions in the elimination of pathogens and activation of immune regulation [37,38]. In our study, we observed a remarkable increase in the plasmic levels of IgD and IgM in gibel carp following CyHV-2 infection. These results suggest that gibel carp responds positively to CyHV-2 infection and generates a protective immune regulation against CyHV-2 invasion through the activated acquired immune response.

In teleost fishes, TLR2 has been minutely researched to identify its function as a PRR for bacterial microbes [39,40]; however, few investigations have been performed about the role of TLR2 in the recognition of viruses, especially viruses with a dsDNA genome [1,22]. Since previous studies involved only TLR2 response to CyHV-2 infection at a certain point of time or in specific tissues (mainly spleen and head kidney), the response of TLR2 to CyHV-2 in the whole infection cycle and multiple tissues (brain, gill, intestine, liver, kidney, and spleen) is unknown. In our study, TLR2 transcripts significantly overexpressed in the kidney and spleen throughout the whole CyHV-2 infection cycle, but overexpressed notably in the brain and gill at 2–7 dpi during CyHV-2 infection. During CyHV-2 infection, the mRNA expression of TLR2 in the liver and intestine upregulated at 1–3 dpi and 3–5 dpi, respectively. TLR2 mRNA expression remained consistently upregulated in six tissues after infection with CyHV-2, though it varied across tissues and at different times. A similar observation was recorded in grass carp (*Ctenopharyngodon idella*) after infection with grass carp reovirus [41]. Besides viral infection, the expression of TLR2 was significantly induced by lipopolysaccharide (LPS) and *Aeromonas hydrophila* in the head kidney, liver, gill, trunk kidney, and spleen of Qihe crucian carp (*Carassius auratus*), indicating that various PRRs could trigger TLR2-mediated immune response in main tissues of fish [39]. The upregulated expression of TLR2 mRNA in six determined tissues indicates that TLR2 could conduct immune recognition to CyHV-2 invasion and virus identification in a time-dependent and tissue-specific manner, thereby inducing immune response in multiple tissues. The virion of CyHV-2 is constitutive of two components: the glycoprotein-embedded lipid envelope and an icosahedral capsid possessing double-stranded DNA. On the basis of proven TLRs’ ligands in other vertebrates, viral glycoproteins have been implicated as a ligand for TLR2 [11]. This suggests that TLR2 might be a receptor for the viral glycoproteins of CyHV-2. Previous studies have shown that the proliferation and assembly of CyHV-2 virions are conducted in hematopoietic cells of the spleen and kidney of infected fish [42]. Hence, this could partly explain the consecutive overexpression of TLR2 transcripts in the kidney and spleen after CyHV-2 infection.

TLR9, a major PRR of vertebrates, can sense unmethylated CpG DNA and trigger the immune response to viral and bacterial DNA. It has been investigated how fish TLR9 responds to the stimulation of bacteria, viruses, and synthetic ligands, such as *A. hydrophila* in *Pelteobagrus vachelli* [43], nervous necrosis virus (NNV) in *Epinephelus coioides* [44], and LPS in *Oreochromis niloticus* [45]. However, little is known about the response of TLR9 to DNA viruses in fish. Herein, this study aims to investigate the expression of TLR9 infected with dsDNA virus (CyHV-2). The mRNA expression levels of TLR9 elevated notably in the spleen throughout the experiment. Additionally, TLR9 transcripts overexpressed observably in the brain, gill, and kidney at four out of seven sampling time points after infection with CyHV-2. After infection with CyHV-2, the relative expression level of TLR9 mRNA dramatically upregulated in the intestine at 5 dpi and in the liver at 1–3 dpi. Similar findings have been reported in other fish species [19,46]. These results imply that TLR9 is involved in the immune response induced by the dsDNA virus in a spatiotemporal manner. Unlike CyHV-2 infection, the expression level of TLR9 is downregulated in the intestine, but upregulated in the liver, spleen, and kidney of *O. niloticus* [45]. This suggests that there is a noticeable variation in TLR9 expression levels across tissues, possibly due to their unique immunological characteristics. However, the underlying mechanism of differential transcriptional regulation of TLR9 among various fish tissues needs further investigation.

In our study, TLR2 and TLR9 exhibited differential expression patterns in immune-related tissues (such as the gill, spleen, intestine, and kidney) following viral challenge by CyHV-2, indicating their respective roles in the immune response to the invading virus (CyHV-2). Our time-based experiment also demonstrated the immediate effect of TLR2 and TLR9 (as early as 1 dpi, remaining elevated until 7 dpi) on controlling viral invasion in the spleen of gibel carp, highlighting the rapid and potent antiviral role of two receptors. The localization of TLRs on the cell surface or in intracellular compartments is crucial for ligand recognition and evokes different and more complex immune recognition of TLRs [47]. Intriguingly, except in the liver, the maximum fold change of TLR2 is much higher than that of TLR9 in the other five tissues. This implies that TLR2 has a stronger immune recognition effect after CyHV-2 infection when compared to TLR9. Therefore, the different expression patterns of TLR2 and TLR9 in various tissues might impact the pathogenesis and outcome of HVHND. A previous study has reported that *Ictalurus punctatus* TLR2 transcripts increased in the spleen after *Ichthyophthirius multifiliis* infection, but its TLR9 transcripts decreased in the spleen after *I. multifiliis* infection, demonstrating discrepant roles of TLR2 and TLR9 against pathogens infection [48]. Differences in TLR location, ligand affinity, and immune status of tissues could have contributed to the compartmentalized and spatiotemporal activation of TLR2- and TLR9-triggered initial pathogen identification process in fish. It has been verified that the expression of TLRs activated by viral infection brings about an innate immune response and changes the levels of cytokines involved in the inflammation including interleukin-1 (IL-1) and tumor necrosis factor α (TNFα), eventually leading to inflammatory damage and necrosis of host cells and tissues [49]. Of note, while signaling from intracellularly localized TLRs can cause either antiviral or inflammatory response, signaling from cell-surface-localized TLRs only leads to inflammatory response [47]. Based on our results, the higher overexpression of TLR2 could be a dominant cause of multiple tissue injuries and high mortality in gibel carp post infection with CyHV-2. In addition, the expression levels of TLR2 and TLR9 in fish serve as indicators of its health status and its ability to defend against the viral pathogens’ infection. Furthermore, TLRs are not only employed as vaccine adjuvants but also as therapeutic targets in viral infections. For instance, Class B CpG, a TLR9 agonist, is used as an adjuvant in influenza vaccines [50]. Imiquimod, a TLR7 agonist, is utilized for curing genital and perianal warts caused by human papilloma virus infection [51]. Hence, TLR2 could function as a promising target for the development of immunotherapeutic strategies against HVHND. Although many obstacles remain concerning treating viral diseases of aquatic animals, TLR-based drugs and vaccines seem to be a ray of light to combat aquatic virus infections. In other words, persistent investigation of TLRs will open new avenues in the aspect of drug discovery and the development of improved vaccines, leading to the development of novel therapeutic and preventive strategies against viral diseases in aquatic animals. These advancements would greatly facilitate the development and improvement of the aquaculture industry in the near future.

## Figures and Tables

**Table 1:** Primers used in quantitative real-time PCR to detect mRNA expressions.

Gene	Primer	Sequence (5′→3′)	Amplicon Size (bp)	GenBank No.
TLR2	TLR2-F	ACGTTTCTGCAAGCTACGGA	71	KC816575.1
	TLR2-R	TCTTTTCCTCGTCTTCGGGC		
TLR9	TLR9-F	CCAGAGTCATTGGCTGGTGT	158	KC816577.1
	TLR9-R	CAGTACCACAGGTCCCAACC		
18S rRNA	18S-F	ATTTCCGACACGGAGAGG	90	XR_003280668.1
	18S-R	CATGGGTTTAGGATACGCTC		

GenBank No. = GenBank accession number, TLR2 = Toll-like receptor 2, TLR9 = Toll-like receptor 9, 18S rRNA = 18S ribosomal RNA.

**Table 2:** Molecular characterization of Toll-like receptor 2 (TLR2) and Toll-like receptor 9 (TLR9) of gibel carp.

Sequence Features	TLR2	TLR9
Chromosome number	8	16
Location	29807236-29812111	11390400-11400035
Orientation	R	F
mRNA accession number	KC816575.1	KC816577.1
mRNA length	2624	3453
Protein accession number	AGO57934.1	AGO57936.1
Amino acid length	791	1064
Isoelectric point	8.63	8.38
Molecular weight (kDa)	122.2	99.2
Subcellular location	Plasma membrane	Plasma membrane

## Data Availability

The authors confirm that the data supporting the findings of this study are available within the manuscript and table.

## References

[1] Fan Y.D., Zhou Y., Zeng L.B., Jiang N., Liu W.Z., Zhao J.Q., Zhong Q.W. (2018). Identification, structural characterization, and expression analysis of toll-like receptors 2 and 3 from gibel carp (*Carassius auratus gibelio*). Fish Shellfish Immunol..

[2] Thangaraj R.S., Nithianantham S.R., Dharmaratnam A., Kumar R., Pradhan P.K., Gopakumar S.T., Sood N. (2020). Cyprinid herpesvirus-2 (CyHV-2): A comprehensive review. Rev. Aquac..

[3] Luo Y.Z., Lin L., Liu Y., Wu Z.X., Gu Z.M., Li L.J., Yuan J.F. (2013). Haematopoietic necrosis of cultured prussian carp, *Carassius gibelio* (Bloch), associated with Cyprinid herpesvirus 2. J. Fish Dis..

[4] Tang R.Z., Lu L.Q., Wang B.Y., Yu J., Wang H. (2020). Identification of the immediate-early genes of Cyprinid Herpesvirus 2. Viruses.

[5] Davison A.J., Kurobe T., Gatherer D., Cunningham C., Korf I., Fukuda H., Hedrick R.P., Waltzek T.B. (2013). Comparative genomics of carp herpesviruses. J. Virol..

[6] Hedrick R.P., Waltzek T.B., McDowell T.S. (2006). Susceptibility of koi carp, common carp, goldfish, and goldfish × common carp hybrids to Cyprinid Herpesvirus-2 and Herpesvirus-3. J. Aquat. Anim. Health.

[7] Iwasaki A., Medzhitov R. (2010). Regulation of adaptive immunity by the innate immune system. Science.

[8] Rauta P.R., Samanta M., Dash H.R., Nayak B., Das S. (2014). Toll-like receptors (TLRs) in aquatic animals: Signaling pathways, expressions and immune responses. Immunol. Lett..

[9] Arancibia S.A., Beltran C.J., Aguirre I.M., Silva P., Peralta A.L., Malinarich F., Hermoso M.A. (2007). Toll-like receptors are key participants in innate immune responses. Biol. Res..

[10] Akira S., Uematsu S., Takeuchi O. (2006). Pathogen recognition and innate immunity. Cell.

[11] Liao Z., Su J. (2021). Progresses on three pattern recognition receptor families (TLRs, RLRs and NLRs) in teleost. Dev. Comp. Immunol..

[12] Meijer A.H., Gabby-Krens S.F., Medina-Rodriguez I.A., He S.N., Bitter W., Ewa Snaar-Jagalska B., Spaink H.P. (2004). Expression analysis of the toll-like receptor and TIR domain adaptor families of zebrafish. Mol. Immunol..

[13] Tanekhy M. (2016). The role of Toll-like Receptors in innate immunity and infectious diseases of teleost. Aquac. Res..

[14] Kiyoshi T., Tsuneyasu K., Shizuo A. (2003). Toll-like receptors. Annu. Rev. Immunol..

[15] Palti Y. (2011). Toll-like receptors in bony fish: From genomics to function. Dev. Comp. Immunol..

[16] Zhu Z.H., Sun Y.N., Wang R.X., Xu T.J. (2013). Evolutionary analysis of TLR9 genes reveals the positive selection of extant teleosts in Perciformes. Fish Shellfish Immunol..

[17] Tu X., Liu L., Qi X.Z., Chen W.C., Wang G.X., Ling F. (2016). Characterization of Toll-like receptor gene expression in goldfish (*Carassius auratus*) during *Dactylogyrus intermedius* infection. Dev. Comp. Immunol..

[18] Wang K.L., Chen S.N., Huo H.J., Nie P. (2021). Identification and expression analysis of sixteen Toll-like receptor genes, TLR1, TLR2a, TLR2b, TLR3, TLR5M, TLR5S, TLR7-9, TLR13a-c, TLR14, TLR21-23 in mandarin fish *Siniperca chuatsi*. Dev. Comp. Immunol..

[19] Zhang X.T., Zhang G.R., Shi Z.C., Yuan Y.J., Zheng H., Lin L., Wei K.J., Ji W. (2017). Expression analysis of nine Toll-like receptors in yellow catfish (*Pelteobagrus fulvidraco*) responding to *Aeromonas hydrophila* challenge. Fish Shellfish Immunol..

[20] Wei Y.C., Hu S., Sun B.B., Zhang Q.H., Qiao G., Wang Z.S., Shao R., Huang G.Q., Qi Z.T. (2017). Molecular cloning and expression analysis of toll-like receptor genes (TLR7, TLR8 and TLR9) of golden pompano (*Trachinotus ovatus*). Fish Shellfish Immunol..

[21] Wu M., Guo L., Zhu K.C., Guo H.Y., Liu B., Jiang S.G., Zhang D.C. (2018). Genomic structure and molecular characterization of Toll-like receptors 1 and 2 from golden pompano *Trachinotus ovatus* (Linnaeus, 1758) and their expression response to three types of pathogen-associated molecular patterns. Dev. Comp. Immunol..

[22] Mou C.Y., Wang Y., Zhang Q.Y., Gao F.X., Li Z., Tong J.F., Zhou L., Gui J.F. (2018). Differential interferon system gene expression profiles in susceptible and resistant gynogenetic clones of gibel carp challenged with herpesvirus CaHV. Dev. Comp. Immunol..

[23] Chen H.J., Luo D.J. (2023). Application of haematology parameters for health management in fish farms. Rev. Aquac..

[24] Lu J., Lu H.D., Cao G.P. (2016). Hematological and histological changes in prussian carp *Carassius gibelio* infected with Cyprinid Herpesvirus 2. J. Aquat. Anim. Health.

[25] Liang L., Xie J., Chen K., Bing X.W. (2015). Pathogenicity and biological characteristics of CyHV-2. Bull. Eur. Assoc. Fish Pathol..

[26] Dulbecco R. (1952). Production of plaques in monolayer tissue cultures by single particles of an animal virus. Proc. Natl. Acad. Sci. USA.

[27] Lawrence M.J., Raby G.D., Teffer A.K., Jeffries K.M., Danylchuk A.J., Eliason E.J., Hasler C.T., Clark T.D., Cooke S.J. (2020). Best practices for non-lethal blood sampling of fish via the caudal vasculature. J. Fish Biol..

[28] Sudhagar A., El-Matbouli M., Kumar G. (2020). Identification and expression profiling of toll-like receptors of brown trout (*Salmo trutta*) during proliferative kidney disease. Int. J. Mol. Sci..

[29] Wang L., He J., Liang L., Zheng X., Jia P., Shi W., Xie J., Liu H., Xu P. (2012). Mass mortality caused by Cyprinid Herpesvirus 2 (CyHV-2) in Prussian carp (*Carassius gibelio*) in China. Bull. Eur. Assoc. Fish Pathol..

[30] Du-Carrée J.L., Morin T., Danion M. (2021). Impact of chronic exposure of rainbow trout, *Oncorhynchus mykiss*, to low doses of glyphosate or glyphosate-based herbicides. Aquat. Toxicol..

[31] Reyes-Becerril M., Angulo C., Ascencio F. (2015). Humoral immune response and TLR9 gene expression in Pacific red snapper (*Lutjanus peru*) experimentally exposed to *Aeromonas veronii*. Fish Shellfish Immunol..

[32] Shen Y., Lau-Cam C.A. (2019). Taurine enhances the protective actions of fish oil against D-galactosamine-induced metabolic changes and hepatic lipid accumulation and injury in the rat. Taurine 11, Advances in Experimental Medicine and Biology.

[33] Yonar S.M. (2019). Growth performance, haematological changes, immune response, antioxidant activity and disease resistance in rainbow trout (*Oncorhynchus mykiss*) fed diet supplemented with ellagic acid. Fish Shellfish Immunol..

[34] Ayyat M.S., Mahmoud H.K., El-Hais A.M., Abd El-Latif K.M. (2017). The role of some feed additives in fish fed on diets contaminated with cadmium. Environ. Sci. Pollut. Res..

[35] Liu J., Zhang P.J., Wang B., Lu Y.T., Li L., Li Y.H., Liu S.J. (2022). Evaluation of the effects of *Astragalus* polysaccharides as immunostimulants on the immune response of crucian carp and against SVCV in vitro and in vivo. Comp. Biochem. Phys. C.

[36] Zhu L.Y., Nie L., Zhu G., Xiang L.X., Shao J.Z. (2013). Advances in research of fish immune-relevant genes: A comparative overview of innate and adaptive immunity in teleosts. Dev. Comp. Immunol..

[37] Xia H., Wu K., Liu W.J., Wang W.M., Zhang X.Z. (2015). Spatio-temporal expression of blunt snout bream (*Megalobrama amblycephala*) mIgD and its immune response to *Aeromonas hydrophila*. Cent. Eur. J. Immunol..

[38] Bilal S., Lie K., Karlsen O.A., Hordvik I. (2016). Characterization of IgM in Norwegian cleaner fish (lumpfish and wrasses). Fish Shellfish Immunol..

[39] Kong X.H., Tang H.R., Zhu Y.C., Zhang J., Li C.J., Zhao X.L., Pei C., Zhou Y., Zeng L.B. (2023). Molecular characterizations of TLR1 and TLR2 in Qihe crucian carp (*Carassius auratus*) and responses to stimulations of *Aeromonas hydrophila* and TLR ligands. Aquac. Int..

[40] Gao Q.X., Xiao Y.P., Zhang C.J., Min M.H., Peng S.M., Shi Z.H. (2016). Molecular characterization and expression analysis of toll-like receptor 2 in response to bacteria in silvery pomfret intestinal epithelial cells. Fish Shellfish Immunol..

[41] He L.B., Wang H., Luo L.F., Jiang S.H., Liu L.Y., Li Y.M., Huang R., Liao L.J., Zhu Z.Y., Wang Y.P. (2016). Characterization, expression analysis and localization pattern of toll-like receptor 1 (tlr1) and toll-like receptor 2 (tlr2) genes in grass carp *Ctenopharyngodon idella*. J. Fish Biol..

[42] Hedrick R.P., Gilad O., Yun S., Spangenberg J., Marty G., Nordhausen R., Kebus M., Bercovier H., Eldar A. (2000). A herpesvirus associated with mass mortality of juvenile and adult koi, a strain of common carp. J. Aquat. Anim. Health.

[43] Qin C.J., Sun J.X., He Y., Wang J., Han Y.W., Li H.T., Liao X.F. (2019). Diurnal rhythm and pathogens induced expression of toll-like receptor 9 (TLR9) in *Pelteobagrus vachellii*. Fish Shellfish Immunol..

[44] Morcillo P., Esteban M.A., Cuesta A. (2013). Effects of nodavirus (VNNV) infection on gene expression profile in the gilthead seabream cell line SAF-1. Fish Shellfish Immunol..

[45] Gao F.Y., Liu J., Lu M.X., Liu Z.G., Wang M., Ke X.L., Yi M.M., Cao J.M. (2021). Nile tilapia Toll-like receptor 7 subfamily: Intracellular TLRs that recruit MyD88 as an adaptor and activate the NF-κB pathway in the immune response. Dev. Comp. Immunol..

[46] Dong X.Y., Su B.F., Zhou S., Shang M., Yan H., Liu F.Q., Gao C.B., Tan F.H., Li C. (2016). Identification and expression analysis of toll-like receptor genes (TLR8 and TLR9) in mucosal tissues of turbot (*Scophthalmus maximus* L.) following bacterial challenge. Fish Shellfish Immunol..

[47] Lester S.N., Li K. (2014). Toll-like receptors in antiviral innate immunity. J. Mol. Biol..

[48] Zhao F., Li Y.W., Pan H.J., Shi C.B., Luo X.C., Li A.X., Wu S.Q. (2013). Expression profiles of toll-like receptors in channel catfish (*Ictalurus punctatus*) after infection with *Ichthyophthirius multifiliis*. Fish Shellfish Immunol..

[49] Yang Y.C., Ren Y.Q., Zhang Y.T., Wang G.X., He Z.W., Liu Y.F., Cao W., Wang Y.F., Chen S.L., Fu Y.S. (2022). A new cell line derived from the spleen of the japanese flounder (*Paralichthys olivaceus*) and its application in viral study. Biology.

[50] Farooq M., Batool M., Kim M.S., Choi S. (2021). Toll-like receptors as a therapeutic target in the era of immunotherapies. Front. Cell Dev. Biol..

[51] Es-saad S., Tremblay N., Baril M., Lamarre D. (2012). Regulators of innate immunity as novel targets for panviral therapeutics. Curr. Opin. Virol..

